# Laparoscopic and Open Distal Pancreatectomy—An Initial Single-Institution Experience with a Propensity Score Matching Analysis

**DOI:** 10.3390/life15010097

**Published:** 2025-01-14

**Authors:** Irena Plahuta, Žan Šarenac, Medeja Golob, Špela Turk, Bojan Ilijevec, Tomislav Magdalenić, Stojan Potrč, Arpad Ivanecz

**Affiliations:** 1 Clinical Department of Abdominal and General Surgery, University Medical Centre Maribor, Ljubljanska Ulica 5, 2000 Maribor, Slovenia; irena.plahuta@ukc-mb.si (I.P.);; 2 Department of Surgery, Faculty of Medicine, University of Maribor, Taborska Ulica 8, 2000 Maribor, Slovenia

**Keywords:** laparoscopic distal pancreatectomy, splenectomy, postoperative pancreatic fistula, intravenous narcotic analgesia, morbidity, mortality, resection rate

## Abstract

Laparoscopic distal pancreatectomy is a minimally invasive approach for the surgical treatment of neoplasms in the distal pancreas. This study aimed to compare this approach to the open procedure. A retrospective analysis of a prospectively maintained database of 400 pancreatectomies was performed. The laparoscopic distal pancreatectomy group (LDP) was compared to the open distal pancreatectomy group (ODP). A propensity score matching analysis (PSM) was performed. From 2016 to 2023, 108 distal pancreatectomies were carried out, 19 (17.6%) laparoscopically and 89 (82.4%) openly. The conversion rate was 13.6%. The severe morbidity rates were 28.1% in the ODP group, 47.4% in the LDP group, and 15.8% in the ODP-PSM group. The difference between the latter two was statistically significant (*p* = 0.034) due to the high rate of Clavien–Dindo grade 3a complications (42.1% versus 10.5%, *p* = 0.042) in the LDP group. The 90-day mortality rates were 3.3% in the ODP group and 5.3% in the other two groups. The LDP group had a shorter duration of intravenous narcotic analgesia (5 versus 7 days, *p* = 0.041). There was no difference in the R0 resection or postoperative pancreatic fistula rates. Our attention should be drawn to preventing postoperative complications because the oncological outcomes are already comparable with those of the open procedure, and postoperative pain management is promising.

## 1. Introduction

The first successful laparoscopic distal pancreatectomy was reported by Alfred Cuschieri thirty years ago [1]. However, the pancreas was long considered unsuitable for minimal access surgical approaches. Obstacles to the wide implementation of laparoscopic distal pancreatectomy include the difficult exposure of the pancreas in the retroperitoneum, the intimate proximity of major vascular structures, the complex technical nature and high complication profile of these operations, concern over adherence to oncological principles, and challenges in training surgeons to perform these low-volume, complex operations [2].

Minimally invasive surgery is a standard for many gastrointestinal surgical procedures, but pancreatic surgery is considered unique and highly specific. The expertise gained in minimally invasive procedures applied in other gastrointestinal areas does not necessarily provide a sufficient guarantee of good outcomes for pancreatic surgery [3].

The initial reports of technical feasibility and safety evaluations were followed through a large multicenter retrospective cohort study (DIPLOMA) and prospective multicenter (LEOPARD) and single-center (LAPOP) randomized control trials comparing laparoscopic with open distal pancreatectomy [4,5,6,7]. These reports confirmed the feasibility and safety of laparoscopic distal pancreatectomy, which is routine in many medical centers worldwide.

There is growing international interest in moving the field forward in an organized manner. A dedicated session for minimally invasive distal pancreatectomy was held at the first international conference on minimally invasive pancreatic resection in conjunction with the annual meeting of the International Hepato-Pancreato-Biliary Association in 2016 [2,3,4]. The first evidence-based guidelines on minimally invasive pancreatic resection were presented in 2019 [8]. The first internationally validated European guidelines on minimally invasive pancreatic surgery achieved consensus in Brescia, Italy, in 2022, and outcome benchmarks have also been established [9,10,11].

The present single tertiary center research has aimed to demonstrate that the laparoscopic approach is not inferior to the open procedure in the entire patient population with neoplasms in the distal pancreas.

## 2. Materials and Methods

A retrospective review of a prospectively maintained database of all consecutive patients who underwent pancreatic resection at a single tertiary referral center was performed. The first laparoscopic distal pancreatectomy was undertaken in April 2016, and data were collected until December 2023.

The patients’ demographics, preoperative clinical characteristics, intraoperative details, histopathological data, and postoperative outcomes were retrieved from the database.

The approach was mainly the surgeons’ preference, but the selection criteria for the laparoscopic one were applied as proposed, as follows: at the beginning, patients with small, presumably benign tumors in the pancreatic tail were selected. With growing experience, patients with malignant, bigger, and more proximal tumors were selected. Caution is still present when the tumor is large, presumably malignant, locally advanced, near the neck of the pancreas, or close to the celiac trunk and its branches [12].

The surgical technique was applied as follows: for laparoscopic distal pancreatectomy, after general anesthesia, the patient was kept supine and was positioned in a 30–45 right lateral decubitus. The surgeon stood on the patient’s right, while the first assistant and the scrub nurse stood on the patient’s left. Pneumoperitoneum was achieved using an optical trocar (Covidien, Medtronic plc, Minneapolis, MN, USA), and three other ports were placed. A 10-mm 30° telescope (Olympus, Tokyo, Japan) was used for visualization. The gastrocolic ligament was dissected to access the pancreas. Laparoscopic ultrasonography (Esaote, Genoa, Italy) was used to determine the tumor and its relationship to the splenic vessels.

After mobilization of the distal pancreas from the retroperitoneum, the splenic artery and vein were clipped. The pancreas was divided at the neck using an Endo GIA™ 60 mm Articulating Extra Thick Reinforced Reload with Tri-Staple™ 2.0 Technology instrument (Covidien, Medtronic plc, Minneapolis, MN, USA). Then, the splenic ligaments were cut.

The specimen was removed using an endo-bag through the Pfannenstiel incision. Passive drainage was placed at the pancreatic bed through the laparoscopic port site.

An open distal pancreatectomy was performed through a left subcostal incision. The steps of the operation were similar to those of a laparoscopic approach, and the same kind of stapler was used. The splenic artery and vein were separately ligated with a 2-0 polyglactin 910 suture. A drain was placed next to the remnant pancreas and brought out through a separate stab incision. The abdominal wall was closed in layers, and the skin was stapled [13,14].

Only pure laparoscopic distal pancreatectomy with splenectomy was carried out, and no hand-assisted or hybrid procedures were performed. There were no multi-visceral or vascular resections. The operative time was defined as the interval from the incision to the skin suturing. The conversion into an open approach was defined as the requirement for laparotomy at any time of the procedure, except for the extraction of the resected specimen.

Diagnoses were based on the final histopathological reports. The histological surgical margins for malignant lesions were defined as microscopically positive (<1 mm, R1) or negative. R0 resection was defined as complete tumor removal with a clear microscopic margin [15,16].

Postoperative morbidity was graded according to the Clavien–Dindo classification and based on the most severe complication. Grades 1 and 2 indicate minor complications that required medical therapies for treatment. Severe complications encompassed grades ≥ 3a, as follows: Grades 3a and 3b necessitate interventional radiological or surgical procedures, grades 4a and 4b involved organ support, and grade 5 signified mortality [17].

A postoperative pancreatic fistula, post-pancreatectomy hemorrhage, and delayed gastric emptying were based on the criteria established by the International Study Group of Pancreatic Surgery [18,19,20]. Reoperation was defined as any surgical procedure performed before discharge from the hospital or in the first 30 postoperative days [14].

The Numeric Pain Rating Scale [21] was used [21] to assess postoperative pain. It consists of a 0–10 scale, with 0 meaning “no pain” and 10 meaning “the worst pain imaginable” [22].

The length of hospital stay was calculated as the interval from the day of surgery to the day of discharge. Patients were discharged when they tolerated oral fluids and a solid diet without intravenous fluid supplementation requirements, their pain was controlled with oral analgesics, and they were mobile enough to take care of themselves [23,24].

Patients consented before the surgery that their anonymous data could be used for research. Therefore, their records were anonymized and deidentified before analysis. The Institutional Ethics Committee approved the research (UKC-MB-KME-41/24). All methods were performed following the relevant guidelines and regulations.

The patients who underwent distal pancreatectomy were divided into two groups and compared according to the type of approach, as follows: laparoscopic distal pancreatectomy (LDP) and open distal pancreatectomy (ODP) groups. Then, a propensity score matching analysis (PSM) was performed, and the groups were compared again.

All consecutive patients with benign or malignant lesions in the body or tail of the pancreas who underwent laparoscopic distal pancreatectomy were eligible for inclusion in this study. There were no exclusion criteria.

The primary endpoints were intraoperative outcomes (operative time and transfusion required). The secondary endpoints were postoperative outcomes (morbidity, severe morbidity, mortality, pancreas-specific morbidity (postoperative pancreatic fistula, post-pancreatectomy hemorrhage, and delayed gastric emptying), reoperation, intensive care unit admission, high-dependency unit stay, time to oral food intake and stool passing, intravenous narcotics requirement, length of hospital stay, readmission, and incisional hernias).

Oncological outcomes (R0 resection and number of harvested lymph nodes) were analyzed in a subset of patients with pancreatic malignant neoplasms.

IBM SPSS for Windows Version 29.0.0.0 (IBM Corporation, Armonk, NY, USA) was used for statistical computations. Percentages are reported to one decimal place. A *p* value < 0.05 was considered statistically significant.

The categorical variables are displayed as numbers with percentages. The differences between categorical variables were tested using Fisher’s Exact test. When more than two categories were present, the Fisher–Freeman–Halton test was used. Continuous variables were expressed as median (interquartile range) and analyzed with the Mann–Whitney test since the distribution analysis showed a non-normal data distribution.

PSM was used to minimize selection bias [25,26]. The patients were matched using relevant variables to equate the complexity of surgical cases. A matched group of patients (ODP-PSM group) was created with a 1:1 ratio without replacement. Standardized mean difference (SMD) was used to assess the balance of the clinical backgrounds between the two groups. An SMD < 0.2 indicated very small differences between the means (optimal balance regarding a variable was generally achieved), an SMD between 0.2 and 0.8 indicated medium differences (a fairly sufficient balance regarding a variable was generally achieved), and an SMD > 0.8 indicated considerable differences (poor balance regarding a variable was generally achieved) [14].

After PSM, the statistical analysis of continuous variables was performed with the Wilcoxon signed-ranks test, and the analysis of categorical variables was performed with the Related-samples Cochran’s Q test and with Related-samples marginal homogeneity test when more than two categories were present [25,27,28].

## 3. Results

**Figure 1:**
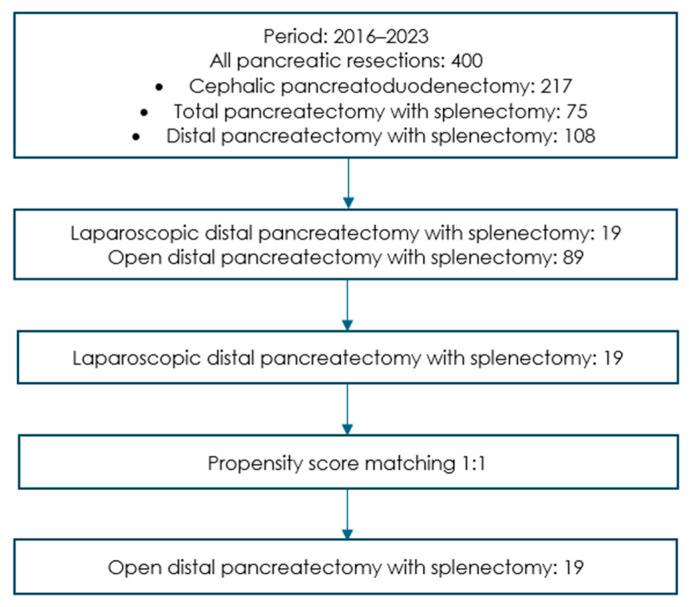
The study flowchart of pancreatectomies from 2016 to 2023.

Figure 1 shows the study flowchart. Our institution performed 400 pancreatic resections from January 2016 to December 2023, including 108 distal pancreatectomies with splenectomies.

**Figure 2:**
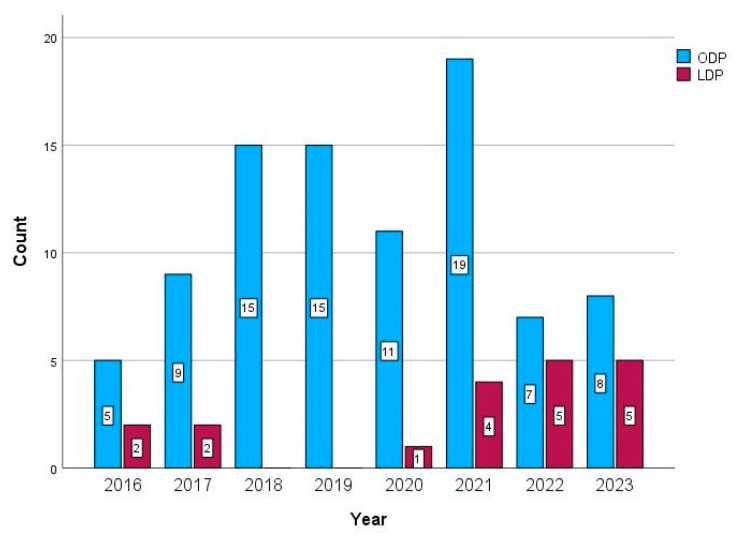
The chronology of distal pancreatectomies at our institution. LDP = laparoscopic distal pancreatectomy; ODP = open distal pancreatectomy.

Our dedicated hepato-pancreato-biliary team consisted of five surgeons who performed open pancreatic resections. Three of them, who were also experienced laparoscopic liver and gastric surgeons, were deployed into the laparoscopic distal pancreatectomy program [29,30,31]. The chronology of the distal pancreatectomy is given in Figure 2.

From the laparoscopic group, 3 (13.6%) cases required conversion to an open procedure, while 19 (86.4%) cases were completed as planned.

The characteristics of LDP versus ODP before and after PSM analysis are summarized in Table 1, Table 2 and Table 3. Table 1 shows the preoperative characteristics of patients and their tumors.

For the PSM, the method was closest to the neighborhood method, with a caliper width of 0.20. The relevant variables were patient-related (sex, age, body mass index, American Society of Anesthesiologists (ASA) score, previous abdominal surgery, and C-reactive protein) and tumor-related (malignancy, tumor size, and tumor site). A matched group of patients was created with a 1:1 ratio (13, 21). The standardized mean difference (SMD) was 0.21. Table 2 shows the outcomes of surgery.

**Figure 3:**
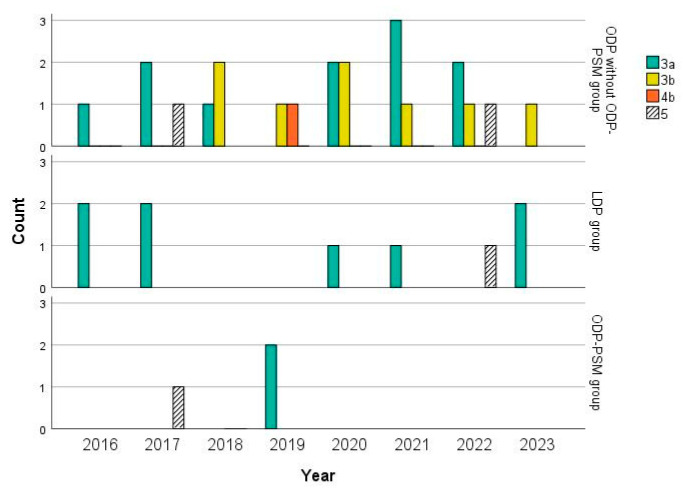
The time-trend analysis of complications of Clavien–Dindo grades ≥ 3a among groups (concordant with Table 2). LDP = laparoscopic distal pancreatectomy; ODP = open distal pancreatectomy; PSM = propensity score matching.

The difference between the LDP and ODP groups was significant when comparing the overall complications; moreover, the difference was due to complications of Clavien–Dindo grade 3a. These also contributed to the significant difference between the LDP and ODP-PSM groups regarding severe complications. Table 3 provides their basic overview, but percentages are not given because one patient can have more than one complication. The time-trend analysis of complications of Clavien–Dindo grades ≥ 3a among groups is given in Figure 3. The 90-day mortality rate was 3.7%. The causes of death are given in Table 3.

The oncological results are given in Table 4.

## 4. Discussion

This research has shown some significant differences between the LDP and ODP groups. The first was a statistically significant difference in the overall postoperative complication rate (*p* = 0.018). It was due to complications of Clavien–Dindo grade 3a, which was 42.1% in the LDP group and 14.6% in the ODP group. The rate in the ODP-PSM group was 10.5%, and the difference was significant (*p* = 0.042). This grade of complications also contributed to the significant difference (*p* = 0.034) between the LDP (42.1%) and ODP-PSM groups (14.6%) in terms of severe complications. The next difference was a shorter duration of intravenous narcotic analgesia (5 vs. 7 days, *p* = 0.041) in the LDP group, which became significant after PSM.

The present study was designed to provide a comprehensive picture of the current laparoscopic distal pancreatectomy in a single tertiary referral center, where the first procedure was performed in 2016. Along with many studies that dealt mainly with pancreatic ductal adenocarcinoma or nonfunctioning pancreatic neuroendocrine neoplasms, our cohort comprises patients with all kinds of diseases that can arise in the distal pancreas [5,14,32,33,34,35].

The clinical characteristics of patients in the LDP and ODP groups did not differ (Table 1), as also reported by Casadei et al., where the differences among the groups became insignificant after PSM [14]. Along with proficiency with open pancreatic resections, the equable selection of patients in both groups could be assigned to the surgeons’ broad experience from laparoscopic liver and gastric surgery [29,36,37]. However, our rate of conversions into the open approach was 13.6%. It was close to the median value of 12.3% in the study by Giani et al., while the benchmark is 2.5% [11] (Table 5). This could be explained by an unfinished learning curve that demands the completion of 25 laparoscopic distal pancreatectomies to gain individual expertise [30,38].

Nevertheless, these three patients were included in the ODP group because the intention-to-treat principle, essential in randomized controlled trials, is more difficult to apply to a retrospective clinical study comparing the outcomes of surgical approaches [39]. One patient had a percutaneous drainage of a postoperative pancreatic fistula (Clavien-Dindo grade 3a) and none of them was not included in the ODP-PSM group.

The primary endpoints were the duration of the operation and the transfusion requirement. Our study showed no differences between the LDP and ODP groups for both outcomes. The median duration of the laparoscopic operation was 202 min, the median in the study by Giani et al. was 232.5 min, and the benchmark was 160 min. The benchmark for intraoperative transfusion application was 0.5% [11]. Our intraoperative blood transfusion rate was 5.3%, without any significant difference with the ODP group (Table 2). This could be explained by a quarter of ASA score III patients and their median age of 69. These patients are prone to bleeding due to tissue fragility [40]. However, more attention should be given to the preoperative anemia correction [41].

This research showed that a shorter duration of intravenous narcotic analgesia (5 vs. 7 days, *p* = 0.041) in the LDP group became significant after PSM (Table 2). Less pain is, apart from the cosmetic effect, the desired consequence of reduced trauma of the abdominal wall [14]. For the assessment of postoperative pain, the Numeric Pain Rating Scale was used [21]. It contained a 0–10 scale, with 0 meaning “no pain” and 10 meaning “the worst pain imaginable” [22]. The intravenous narcotic, namely piritramide, was administered when the patients estimated their pain ≥ 6.

It is proposed that narcotic analgesia can contribute to the occurrence of postoperative pancreatic fistula after distal pancreatectomy due to the spasm of the sphincter of Oddi, which increases pressure within the pancreatic duct stump [42,43]. However, pancreas-specific morbidity was insignificant between the groups (Table 2). For postoperative pancreatic fistula type B, our rates in the ODP group were 28.1%, 36.8% in the LDP group, and 26.3% in the ODP-PSM group. Postoperative pancreatic fistula type C was observed only in open distal pancreatectomies. The benchmark for fistula type B/C was set at 8.3% [11].

In 2021, our institution unified the surgical technique, where one type of stapler is used in open and laparoscopic distal pancreatectomies. The staplers are proven useful when the pancreatic thickness is <12 mm [44,45,46]. Recently, has been suggested that a pre-firing pancreatic compression for at least 10 min might reduce the risk of clinically relevant postoperative pancreatic fistula development, especially when a pancreatic gland tissue is thick [47,48].

Among the secondary endpoints, our overall morbidity rate was 76.4% in the ODP group, 94.7% in the LDP group, and 68.4% in the ODP-PSM group. Both comparisons were statistically significant (Table 2). This skyrocketing rate in the LDP group was due to the complications of Clavien–Dindo grade 3a (42.1%), which signified percutaneous drainages of fluid collections in the thorax or abdomen (Table 3). They also contributed to the statistical significance of the severe morbidity rates (Clavien–Dindo ≥ 3a) in this group (47.4%). However, this rate was insignificant compared to the ODP group (28.1%) but showed significance when compared to the ODP-PSM group (10.5%; *p* = 0.034). The benchmark was 8.4% [11]. The proposed risk factors for severe morbidity were ASA grade > II, multi-visceral resections, and robotic resections [11]. However, one-quarter of our patients were ASA grade III, but there were no multi-visceral or robotic resections. As can be observed in Figure 2 and Figure 3, the count of LDP had a growing trend, and the count of its severe complications had a decreasing one. Of note, leaving the drain in place for sufficient time could diminish the rate of complications of Clavien–Dindo grade 3a, since the rates of postoperative pancreatic fistula type B were insignificant among the groups.

Mortality rates, reoperations, and readmissions did not differ between the LDP and ODP groups (Table 2). The 90-day mortality rate of 3.7% in the whole cohort was caused by medical complications in three patients (Table 3). One patient died of irreversible septic shock following a toxic megacolon due to a postoperative pancreatic fistula. Perioperative mortality close to zero has always been a desired benchmark (Table 5) [11]. On the other hand, the rate of reoperations was 0 in the LDP group and 9% in the ODP group. The reasons for reoperations are given in Table 3.

The duration of a high-dependence unit or hospital stay depends on many factors, including the availability of personnel, facilities, and social and cultural factors, which differ between wards and lands [49]. Nevertheless, there was no difference in these variables among our groups; moreover, the LDP group (nine days) was close to the median of the duration of the hospital stay in this study (eight days). Furthermore, a readmission rate is proposed as a more unified outcome, and the time to functional recovery may become a replacement outcome for the duration of a hospital stay [11]. The readmission rates were indifferent among the groups; furthermore, the LDP group had a rate of 15.8%, while the median was 13%, and the benchmark was 4.1%. There was no difference between the groups in the functional recovery of patients, which included four days to oral food intake and three days to stool passing in both groups after PSM. Therefore, a benchmark of five days of hospital stay is obtainable (Table 2 and Table 5).

Furthermore, the oncological outcomes were analyzed in a subset of patients with pancreatic malignant neoplasms. As shown in Table 4, there were no differences between the LDP and ODP groups. After PSM, the rate of R0 resections was 84.6% in the LDP group and 90.9% in the matched group. In 2014, the International Study Group on Pancreatic Surgery set guidelines for lymphadenectomy in pancreatic ductal adenocarcinoma. For tumors of the pancreatic body and tail, removal of stations 10, 11, and 18 was set as the standard, also because of adequate nodal staging [50]. The first and second echelons were recently established in pancreatoduodenectomy [16]. Several research studies have confirmed the importance of those stations for distal pancreatectomy, adding that the optimal extent of lymphadenectomy should be estimated based on the tumor location [51,52,53]. The analysis by Zhun Hong Wong et al. showed no significant difference between laparoscopic, robotic, and open distal pancreatectomies for harvested lymph nodes, resection margins, and tumor recurrence; however, the patients’ survival rate was better in laparoscopic and robotic groups when compared to the open resection group [33].

Moreover, Partelli et al. published a study on nonfunctioning pancreatic neuroendocrine neoplasms, in which the rate of severe postoperative morbidity was lower and the duration of hospital stay was shorter in the laparoscopic group. Despite the significantly lower number of harvested lymph nodes in the laparoscopic group (13 versus 10, *p* = 0.0036), similar disease-free and overall survival rates were reported for both groups [35].

The present study has several limitations. First, the number of patients in the LDP group is relatively low, which can lead to the loss of statistical power. Second, given the retrospective nature of this study, selection bias may have been present. Although propensity score matching was performed to overcome potential bias and make the two groups similar, it is less effective than a prospective randomized trial. Another important issue is the failure to meet many of the international benchmarks. Therefore, a future clinical direction will be to increase the volume of LDP in order to overcome the challenges of the learning curve. Future research aims to compare the long-term survival rates of patients with malignant tumors in the distal pancreas.

To conclude, our attention should be drawn to preventing postoperative complications, because the oncological outcomes are already comparable with those of the open procedure, and postoperative pain management is promising. Much will have to be done to achieve the benchmark values.

## Figures and Tables

**Table 1:** Preoperative characteristics of 108 patients and their tumors before and after a propensity score matching analysis of LDP versus ODP.

Variable	Overall Analysis				Propensity Score Matched Patients		
	All Patients 108 (%)	LDP (*n* = 19, 17.6%)	ODP (*n* = 89, 82.4%)	*p* Value	LDP (*n* = 19, 100%)	ODP-PSM (*n* = 19, 100%)	*p* Value
Male sex	50 (46.3)	8 (42.1)	42 (47.2)	0.802 ^a^	8 (42.1)	7 (36.8)	0.705 ^b^
Age (years)	66 (19)	69 (14)	64 (19)	0.085 ^c^	69 (14)	66 (13)	0.090 ^d^
Body mass index (kg/m^2^)	27.3 (8.2)	26.7 (8.9)	27.3 (8)	0.623 ^c^	26.7 (8.9)	27.6 (7.4)	0.717 ^d^
ASA score III	27 (25.0)	5 (26.3)	22 (24.7)	1.000 ^a^	5 (26.3)	5 (26.3)	0.480 ^b^
Comorbidities present	75 (69.4)	14 (73.7)	61 (68.5)	0.787 ^a^	14 (73.7)	14 (73.7)	1.000 ^b^
Number of comorbidities	2 (2)	2 (2)	2 (3)	0.663 ^c^	2 (2)	2 (4)	0.931 ^d^
Previous abdominal surgery	42 (38.9)	11 (57.9)	31 (34.8)	0.073 ^a^	11 (57.9)	9 (47.4)	0.480 ^b^
C-reactive protein > 5 mg/L	28 (25.9)	4 (21.1)	24 (27.0)	0.775 ^a^	4 (21.1)	5 (26.3)	0.705 ^b^
Carcinoembryonic antigen > 5 μg/L	17 (15.7)	1 (5.3)	16 (18.0)	0.297 ^a^	1 (5.3)	4 (21.1)	0.180 ^b^
Carbohydrate antigen 19-9 > 37 kU/L	42 (38.9)	7 (36.8)	35 (39.3)	1.000 ^a^	7 (36.8)	7 (36.8)	1.000 ^b^
Malignant tumors	69 (63.9)	13 (68.4)	56 (62.9)	0.794 ^a^	13 (68.4)	11(57.9)	0.414 ^b^
Tumor size (mm)	35 (27)	29 (26)	35 (25)	0.124 ^c^	29 (26)	35 (25)	0.121 ^d^
Tumor in the neck/body	57 (52.8)	7 (36.8)	50 (56.2)	0.138 ^a^	7 (36.8)	5 (26.3)	0.480 ^b^

^a^ Categorical variable reported as *n* (%), Fisher’s Exact test and ^b^ the Related-samples Cochran’s Q test; ^c^ continuous variable, non-normal distribution, reported as median (interquartile range), Mann-Whitney test and ^d^ Wilcoxon signed-ranks test. ASA = American Society of Anesthesiologists; LDP = laparoscopic distal pancreatectomy; ODP = open distal pancreatectomy; PSM = propensity score matching.

**Table 2:** Outcomes of 108 patients: intraoperative outcomes, recovery, morbidity, and mortality before and after a propensity score matching analysis of LDP versus ODP.

Outcome	Overall Analysis				Propensity Score Matched Patients		
	All Patients *n* (%)	LDP (*n* = 19, 17.6%)	ODP (*n* = 89, 82.4%)	*p* Value	LDP (*n* = 19, 100%)	ODP-PSM (*n* = 19, 100%)	*p* Value
Operative time (min)	190 (80)	202 (110)	185 (90)	0.084 ^c^	202 (110)	190 (140)	0.602 ^d^
Blood loss (mL)	209 (241)	134 (64)	226 (279)	0.073 ^c^	134 (64)	245 (330)	0.121 ^d^
Intraoperative blood transfusion	6 (5.6)	1 (5.3)	5 (5.6)	1.000 ^a^	1 (5.3)	1 (5.3)	1.000 ^b^
No complications	22 (20.4)	1 (5.3)	21 (23.6)	0.018 ^e^	1 (5.3)	6 (31.6)	0.042 ^f^
Clavien–Dindo 1	25 (23.1)	7 (36.8)	18 (20.2)		7 (36.8)	4 (21.1)	
Clavien–Dindo 2	27 (25.0)	2 (10.5)	25 (28.1)		2 (10.5)	6 (31.6)	
Clavien–Dindo 3a	21 (19.4)	8 (42.1)	13 (14.6)		8 (42.1)	2 (10.5)	
Clavien–Dindo 3b	8 (7.4)	0 (0.0)	8 (9.0)		0 (0.0)	0 (0.0)	
Clavien–Dindo 4b	1 (0.9)	0 (0.0)	1 (1.1)		0 (0.0)	0 (0.0)	
Clavien–Dindo 5	4 (3.7)	1 (5.3)	3 (3.3)		1 (5.3)	1 (5.3)	
Severe morbidity (Clavien–Dindo ≥ 3a)	34 (30.6)	9 (47.4)	25 (28.1)	0.111 ^a^	9 (47.4)	3 (15.8)	0.034 ^b^
30-day mortality	1 (0.9)	0 (0.0)	1 (1.1)	1.000 ^a^	0 (0.0)	1 (5.3)	0.317 ^b^
90-day mortality	4 (3.7)	1 (5.3)	3 (3.3)	0.444 ^a^	1 (5.3)	1 (5.3)	1.000 ^b^
Post-pancreatectomy hemorrhage (all three grades)	3 (2.8)	0 (0.0)	3 (3.4)	1.000 ^a^	0 (0.0)	1 (5.3)	0.317 ^b^
Delayed gastric emptying	2 (1.9)	1 (5.3)	1 (1.1)	0.322 ^a^	1 (5.3)	1 (5.3)	1.000 ^b^
Postoperative pancreatic fistula type B	32 (29.6)	7 (36.8)	25 (28.1)	0.580 ^a^	7 (36.8)	5 (26.3)	0.527 ^b^
Postoperative pancreatic fistula type C	8 (7.4)	0 (0.0)	8 (9.0)	0.346 ^a^	0 (0.0)	1 (5.3)	0.317 ^b^
Reoperation	11 (10.2)	1 (5.3)	10 (11.2)	0.685 ^a^	1 (5.3)	0 (0.0)	0.317 ^b^
Readmission	23 (21.3)	3 (15.8)	20 (22.5)	0.759 ^a^	3 (15.8)	1 (5.3)	0.317 ^b^
Hospital stay (days)	11 (7)	9 (7)	11 (8)	0.074 ^c^	9 (7)	12 (11)	0.538 ^d^
Intensive care unit admission	7 (6.5)	1 (5.3)	6 (6.7)	1.000 ^a^	1 (5.3)	1 (5.3)	1.000 ^b^
High-dependency unit stay (days)	5 (2)	5 (3)	5 (2)	0.849 ^c^	5 (3)	5 (2)	0.451 ^d^
Time to oral food intake (days)	4 (2)	4 (2)	4 (2)	0.878 ^c^	4 (2)	4 (3)	0.647 ^d^
Time to stool passing (days)	4 (1)	3 (1)	4 (1)	0.866 ^c^	3 (1)	3 (2)	0.523 ^d^
Intravenous narcotics requirement (days)	6 (3)	5 (4)	6 (3)	0.214 ^c^	5 (4)	7 (2)	0.041 ^d^
Incisional hernia	14 (13.0)	2 (10.5)	12 (13.5)	1.000 ^a^	2 (10.5)	3 (15.8)	0.655 ^b^

^a^ Categorical variable reported as *n* (%), Fisher’s Exact test and ^b^ the Related-samples Cochran’s Q test; ^c^ continuous variable, non-normal distribution, reported as median (interquartile range), Mann-Whitney test, and ^d^ Wilcoxon signed-ranks test; ^e^ categorical variable with more than two groups, reported as *n* (%), Fisher–Freeman–Halton test and ^f^ Related-samples marginal homogeneity test. LDP = laparoscopic distal pancreatectomy; ODP = open distal pancreatectomy; PSM = propensity score matching.

**Table 3:** An overview of severe complications.

	ODP Without ODP-PSM Group	LDP Group	ODP-PSM Group
Clavien–Dindo 3a (intervention without general anesthesia)			
Thoracic drainage		1	
Postoperative pancreatic fistula drainage	11	6	2
Subphrenic abscess drainage	2	1	
Gastric bleeding–endoscopic hemostasis			1
Clavien–Dindo 3b (intervention under general anesthesia)			
Subtotal colectomy due to toxic megacolon	1		
Reduction in internal hernia	1		
Drainage of the abscess	3		1
Duodenum-preserving pancreatectomy	2		
Hemostasis of bleeding from lienal artery	2		
Partial omentectomy due to necrosis	1		
Lymphorrhea–sutures of cisterna chyli	1		
Colostomy due to the colocutaneous fistula		1	
Clavien–Dindo 4b (multiorgan dysfunction)			
Septic shock due to portal vein thrombosis	1		
Clavien–Dindo 5 (death)			
Pulmonary embolism			1
Acute myocardial infarction	1		
Septic shock	1		
Coronavirus disease 2019 pneumonia		1	

LDP = laparoscopic distal pancreatectomy; ODP = open distal pancreatectomy; PSM = propensity score matching.

**Table 4:** Histopathological reports of 69 (63.9%) patients with malignant tumors before and after a propensity score matching analysis of LDP versus ODP.

Outcome	Overall Analysis				Propensity Score Matched Patients		
	All Patients *n* = 69	LDP (*n* = 13, 68.4%)	ODP (*n* = 56, 62.9%)	*p* Value	LDP (*n* = 13, 68.4%)	ODP-PSM (*n* = 11, (57.9%)	*p* Value
Diagnosis							
Pancreatic ductal adenocarcinoma	39 (56.5)	5 (38.5)	34 (60.7)	0.191 ^e^	5 (38.5)	6 (54.5)	0.564 ^f^
Nonfunctioning pancreatic neuroendocrine neoplasms	27 (39.1)	8 (61.5)	19 (33.9)		8 (61.5)	4 (36.4)	
Other	3 (4.3)	0 (0.0)	3 (5.4)		0 (0.0)	1 (9.1)	
Number of harvested lymph nodes	14 (12)	14 (9)	15 (16)	0.685 ^c^	14 (9)	15 (22)	0.284 ^d^
Resection margin (mm)	3 (8)	4 (11)	3 (9)	0.786 ^c^	4 (11)	6 (15)	0.929 ^d^
R0 resection	57 (82.6)	11 (84.6)	46 (82.1)	1.000 ^a^	11 (84.6)	10 (90.9)	1.000 ^b^

^a^ Categorical variable reported as *n* (%), Fisher’s Exact test, and ^b^ the Related-samples Cochran’s Q test; ^c^ continuous variable, non-normal distribution, reported as median (interquartile range), Mann-Whitney test, and ^d^ Wilcoxon signed-ranks test; ^e^ categorical variable with more than two groups, reported as *n* (%), Fisher–Freeman–Halton test, and ^f^ Related-samples marginal homogeneity test. LDP = laparoscopic distal pancreatectomy; ODP = open distal pancreatectomy.

**Table 5:** Range, median, and benchmark values in minimally invasive distal pancreatectomy with splenectomy at a glance (adopted from Giani et al. [11]).

Outcome	Range	Median Value	Benchmark
Duration of operation (min)	132.5–361.5	232.5	160
Conversion (%)	0–54.5	12.3	2.5
Intraoperative blood transfusion (%)	0–10.8	2.6	0.5
Overall morbidity (%)	25.6–100	58.0	30.4
Severe morbidity (%)	4.4–54.7	17.4	8.4
Postoperative pancreatic fistula (%)	6.3–47.4	21.9	8.3
Reoperations (%)	0–20.0	5.4	1.8
Duration of hospital stay (days)	5–13	8	5
Readmissions (%)	0–40	13.0	4.1
90–days mortality	0–17.6	0	0

## Data Availability

Due to the patients’ privacy, the data presented in this study are available upon request from the corresponding author.
